# Conduct disorder in females is associated with reduced corpus callosum structural integrity independent of comorbid disorders and exposure to maltreatment

**DOI:** 10.1038/tp.2015.216

**Published:** 2016-01-19

**Authors:** P Lindner, I Savic, R Sitnikov, M Budhiraja, Y Liu, J Jokinen, J Tiihonen, S Hodgins

**Affiliations:** 1 Department of Clinical Neuroscience, Karolinska Institutet, Stockholm, Sweden; 2 Centre for Psychiatry Research, Karolinska Institutet, Stockholm, Sweden; 3 Department of Women's and Children's Health, Karolinska Institutet, Stockholm, Sweden; 4 Neurology Clinic, Karolinska University Hospital, Huddinge, Sweden; 5 Department of Clinical Radiology, Kuopio University Hospital, University of Eastern Finland, Kuopio, Finland; 6 Department of Neurology, Kuopio University Hospital, University of Eastern Finland, Kuopio, Finland; 7 Department of Clinical Sciences, Umeå University, Umeå, Sweden; 8 Department of Forensic Psychiatry, University of Eastern Finland, Niuvanniemi Hospital, Kuopio, Finland; 9 National Institute for Health and Welfare, Helsinki, Finland; 10 Département de Psychiatrie, Université de Montréal, Montréal, QC, Canada

## Abstract

The behavioral phenotype and genotype of conduct disorder (CD) differ in males and females. Abnormalities of white matter integrity have been reported among males with CD and antisocial personality disorder (ASPD). Little is known about white matter integrity in females with CD. The present study aimed to determine whether abnormalities of white matter are present among young women who presented CD before the age of 15, and whether abnormalities are independent of the multiple comorbid disorders and experiences of maltreatment characterizing females with CD that may each in themselves be associated with alterations of the white matter. Three groups of women, aged on average 24 years, were scanned using diffusion tensor imaging and compared: 28 with prior CD, three of whom presented ASPD; a clinical comparison (CC) group of 15 women with no history of CD but with similar proportions who presented alcohol dependence, drug dependence, anxiety disorders, depression disorders and physical and sexual abuse as the CD group; and 24 healthy women. Whole-brain, tract-based spatial statistics were computed to investigate differences in fractional anisotropy, axial diffusivity and radial diffusivity. Compared with healthy women, women with prior CD showed widespread reductions in axial diffusivity primarily in frontotemporal regions. After statistically adjusting for comorbid disorders and maltreatment, group differences in the corpus callosum body and genu (including forceps minor) remained significant. Compared with the CC group, women with CD showed reduced fractional anisotropy in the body and genu of the corpus callosum. No differences were detected between the CD and healthy women in the uncinate fasciculus.

## Introduction

In the United States, conduct disorder (CD) affects ~7% of females.^1^ This diagnosis indexes a pattern of antisocial behavior including violation of rules and norms, bullying, theft and physical assault during childhood or adolescence.^2^ CD is a heritable disorder of neural development,^3^ associated with a wide range of negative outcomes through adolescence and adulthood, including persistent antisocial behavior, academic failure, low socioeconomic status, criminality and mental and physical disorders.^4^ Importantly, women who presented CD by mid-adolescence provide non-optimal parenting to their offspring,^5^ as well as susceptibility genes,^6^ thereby contributing to the intergenerational transfer of antisocial behavior.

The CD phenotype differs considerably in males and females. The prevalence of CD is lower in females than in males,^7^ the age of onset is later, the developmental course differs,^8, 9^ as does symptom presentation, with less aggressive behavior among females than males.^10^ Recent evidence suggests that the genotype also differs.^11, 12^ In addition, some of the brain structures associated with antisocial and aggressive behavior differ in males and females,^13, 14^ as does the rate of brain maturation^15, 16^ and the neural mechanisms underlying emotional processing.^17^

Little is known about the neural correlates of CD in females. Available findings suggest the possibility of abnormalities of the white matter architecture. However, with one exception,^18^ all previous studies of white matter among children/adolescents with CD have been carried out using mixed sex or male-only samples. Recent studies using diffusion tensor imaging (DTI)^19^ report abnormalities of the uncinate fasciculus (UF), the principal white matter tract connecting the amygdala and orbitofrontal cortex,^20^ among antisocial males but not females. Boys with CD, compared with healthy boys, showed increased fractional anisotropy (FA) of the UF, in the left hemisphere,^21^ and in both hemispheres,^18, 22^ whereas no differences in the FA of the UF were observed among girls with CD relative to healthy girls.^18^ One study that included teenage boys and girls with CD observed no difference in the FA in the UF or elsewhere,^23^ whereas another reported decreased FA and decreased axial diffusivity (AD) in a widespread frontotemporal area covering most major tracts, including the UF.^24^ Abnormalities of the corpus callosum, detected with DTI, have also been reported among boys and girls with CD,^24^ boys with CD,^25^ and adult males with antisocial personality disorder (ASPD) who, by definition, presented CD before the age of 15.^26^ There has been no prior whole-brain DTI study of females with CD.

Girls with CD, as compared with typically developing girls, present lower than average intelligence quotient (IQ),^27^ higher rates of alcohol- and drug-use disorders (substance use disorders (SUDs)),^1, 28^ anxiety and depression disorders,^29^ and physical and sexual abuse.^30^ Yet, previous neuroimaging studies have either recruited atypical samples of participants with CD who did not present elevated proportions with these comorbid disorders and experiences of maltreatment, or did not take full account of these disorders and experiences when examining abnormalities, thereby limiting the clinical usefulness of the findings. In order to increase the knowledge of brain abnormalities among females with CD that may subsequently inform the development of treatments, it is necessary to distinguish abnormalities associated with CD, those associated with the commonly comorbid disorders and those associated with childhood maltreatment. To do so presents a challenge as IQ, the commonly associated comorbid disorders and experiences of maltreatment are each independently associated with abnormalities of white matter integrity. IQ has been associated with FA of specific tracts, including the UF and corpus callosum.^31^ Both alcohol- and drug-use disorders have been associated with reduced FA across the brain, including the orbitofrontal cortex and the anterior corpus callosum.^32, 33, 34, 35, 36, 37, 38^ Anxiety and depression disorders are associated with reduced FA of the UF.^39, 40, 41, 42, 43, 44, 45^ Physical and sexual abuses have consistently been associated with structural abnormalities of the corpus callosum.^46, 47^

In sum, little is known about white matter abnormalities specifically associated with CD in females. The present study aimed to determine whether young women who had presented CD before the age of 15, as compared with healthy women with no history of conduct problems, mental disorders or maltreatment, present abnormalities of the white matter structure. Further, the study aimed to determine whether any identified abnormalities of the white matter were associated with CD specifically, or with the common comorbid disorders of alcohol dependence, drug dependence, anxiety and depression, and experiences of physical and sexual abuse. The women underwent diffusion-weighted magnetic resonance imaging (MRI). Measures of diffusivity and anisotropy were used as proxy measures of white matter integrity. Two complementary strategies were adopted in order to disentangle the neural correlates of CD, comorbid disorders and experiences of physical and sexual abuse.^48^ One, in comparisons of women with CD and healthy women, lifetime comorbid disorders and histories of physical and sexual abuse were entered into analyses of group differences as covariates. Two, women with CD were compared with a group of women without CD that included similar proportions of participants with comorbid mental disorders and physical and sexual abuse as the CD group. Females with CD exhibit early puberty that is associated with neural development.^15^ All participants were in their mid-twenties, and hence there was no confounding effect of the pubertal development stage.

## Materials and methods

### Ethics

The current study, and all previous waves of data collection, was approved by the Regional Ethical Review Board (Etikprövningsnämnden) in Stockholm.

### Participants

The CD group included 22 women who were initially recruited in 2004 when they sought treatment for SUDs,^49^ and six women recruited from among sisters of the clients at the SUDs clinic (none of whom were related to the other 22 women with CD). The healthy comparison (HC) included 24 women matched for age with the CD group, none with any past or current axis I or II disorder (except two cases who presented past alcohol abuse) nor any criminal activity as juveniles or adults, nor any experience of physical or sexual abuse. The study was powered to detect a large (Cohen's *d*=0.8) difference in cluster-average diffusivity and anisotropy metrics between the CD (*n*=28) and HC groups (*n*=24) with *t*-tests and 80% power. The clinical comparison (CC) group included 15 women without CD (maximum of one lifetime CD symptom) who were recruited from the same SUDs clinic as those with CD. They presented similar proportions with diagnoses of alcohol dependence, drug dependence, anxiety disorders, depression disorders and experiences of maltreatment as participants with CD. No participant had a history of bipolar disorder, psychosis, physical handicap or neurological disorder, and all met standard MRI inclusion criteria.

### Procedure

The CD and CC groups were contacted by mail and by telephone and invited to participate in a brain imaging study. The HC women responded to announcements placed on company bulletin boards and on the internet. On the telephone, the study was explained to participants, and they were screened to ensure eligibility for the study and MRI. Participants were asked to refrain from alcohol and drug use for 3 days before participation. Upon arriving at the laboratory, they signed informed consents to participate, and completed a diagnostic interview and self-report questionnaires. As the HCs were new to the study, they additionally completed an intelligence test. Before the scan, participants used a breathalyzer and provided saliva to test for recent use of alcohol and drugs. None tested positive. Participants were compensated with 1600 Swedish Kronor in gift certificates.

### Measures

The CD and CC participants were first assessed in mid-adolescence,^49^ when those aged 18 years or younger completed the Schedule for Affective Disorders and Schizophrenia for School-Age Children,^50^ and those aged 18 or older completed the Structured Clinical Interview for DSM IV (SCID I and II).^51^ They were re-assessed 6, 12 and 60 months later.^52, 53^ Approximately 18 months after the last reassessment, the scanning study began. Before the scan, the HC women completed the SCID I and II, and the CD and CC women completed only the SCID I. The revised Conflict Tactics Scales^54^ were used to assess physical abuse in childhood; the Sexual and Physical Abuse Questionnaire,^55^ the Sexual Experiences Survey^56^ and the MacArthur Community Violence Instrument^57^ were used to assess sexual abuse. The MacArthur community violence instrument was used to assess aggressive behavior during 6 months before the brain scan. Participants also provided information on current psychosocial functioning. The presence of attention-deficit hyperactivity disorder (ADHD) was indexed by a diagnosis or methylphenidate medication as reported by participants at any past interview, and for the CD and CC groups in the Swedish national health register. Verbal and performance intelligence (VIQ and PIQ) were estimated using the Vocabulary and Block design subtests of the Wechsler Adult Intelligence Scale III,^58^ either before scanning (HC group) or at past assessments (CD and CC groups).^49, 52, 53^ Handedness was assessed using the self-rated Edinburgh Handedness Inventory before scanning.^59^ See Supplementary Table S1 for details on measurements.

### MRI acquisition

Scanning was completed in a single session with a 3-Tesla MRI scanner (MR750 GE Healthcare, Milwaukee, WI, USA). A single-shot echo planar imaging, twice-refocused spin-echo diffusion pulse sequence was enhanced by slice-selective gradient reversal to improve fat suppression.^60^ DTI data were acquired across 60 noncollinear directions ordered using electrostatic repulsion algorithm^61^ with *b*=1000 s mm^−2^ along with eight initial b0 directions. Field-of-view was 23 cm, acquisition matrix 116 × 116 and slice thickness 2 mm, providing 2-mm isotropic resolution. Echo time was 81.6 ms and repetition time 7600 ms. Data were acquired with an eight-channel array coil (*in-Vivo*, Gainesville, FL, USA). Preprocessing of images was performed using DTIPrep^62^ and the FSL software package.^63, 64^ For details, see Supplementary Methods.

### Statistical analyses

Sociodemographic and clinical characteristics of the CD, HC and CC groups were compared pairwise in the R (3.2) statistical environment using F-tests, Wilcoxon rank sum test and Fisher's exact tests. Preprocessed DTI data were analyzed using Tract-Based Spatial Statistics (TBSS).^65^ FA images of all subjects were aligned into FMRIB58 standard space using the FNIRT nonlinear registration tool. A mean FA image was created, from which a tract skeleton was generated using the standard lower threshold of 0.2. Each participant's aligned FA map was then projected onto this skeleton. As FA is an unspecific marker of abnormality, AD and radial diffusivity (RD) were also analyzed. As is recommended, FA images were used to achieve registration, skeletonization and estimation of projection vectors for AD and RD maps. Separate voxelwise comparisons of FA, AD and RD maps were computed using nonparametric statistics (the *randomise* tool) with threshold-free cluster enhancement and 5000 permutations per contrast. All reported TBSS results were corrected for multiple comparisons using familywise error (FWE) correction, contrastwise. Because of the three possible pairwise group contrasts, we further corrected for multiple comparisons by considering FWE-corrected *P*-values below 0.017 (0.05/3), significant in each statistical contrast. Mask-average FA, AD and RD values were extracted using the *fslmeants* tool, exported to R and analyzed using F-tests and Pearson's correlation coefficients (statistical assumptions checked in each contrast; Spearman's correlations used for handedness data due to distribution). Cohen's *d* effect sizes (with bootstrapped confidence intervals) were calculated in R based on group means and s.d.'s for the mask-average values.

Initially, CD and HC groups were compared by conducting whole-brain, voxelwise analyses of FA, AD and RD maps. The statistical map of the one significant contrast was thresholded and binarized to include only voxels with *P*_FWE_<0.017, creating a mask. In step two, using this mask, the same statistical contrast was re-run six times, each time with a different covariate (lifetime history of alcohol dependence, drug dependence, anxiety disorder, depression disorder, physical abuse and sexual abuse). The six resulting statistical maps were then thresholded and binarized. In contrast with a statistical model including all covariates simultaneously, this strategy allowed calculation of the percentage of previously significant voxels that were lost when adding each covariate, and also avoided statistical issues of collinearity and possible interactions among the covariates. A conjunction mask was created by multiplying the six binarized statistical maps, creating a mask of voxels that remained significant after controlling for every covariate, and was used to extract mask-average values.

In a final step, voxelwise comparisons of FA, AD and RD maps of the CD and CC groups were computed. Owing to the clinical similarities between the CD and CC groups, between-group effect sizes were expected to be smaller than those of CD and HC. Therefore, to increase power, this analysis was restricted to a region-of-interest corresponding to the conjunction mask hypothesized to be a region of abnormal integrity specifically associated with CD. As before, significant voxels were used to create masks and average values exported to R.

## Results

Whole-brain voxelwise analyses revealed no significant (*P*_FWE_<0.017) correlations between the AD, FA or RD values of any voxel with age, handedness, VIQ or PIQ.

### Women with CD compared with healthy women

As shown in Table 1, women with CD were similar to HC women as to age and handedness. They presented lower VIQ (*P*=0.009), a trend toward lower PIQ (*P*=0.069) and significantly fewer had completed high school. Proportionately, a greater number of women with CD than the HC women obtained lifetime diagnoses of alcohol dependence, drug dependence, anxiety disorders as well as depression disorders, and reported physical abuse by parents and sexual abuse. In adulthood, the women with CD, as compared with HC, showed lower levels of psychosocial functioning, more aggressive behavior and proportionately more were mothers, and only three presented ASPD. At time of the scan, few presented alcohol or drug dependence, or depression, whereas 29% presented an anxiety disorder.

**Figure 1:**
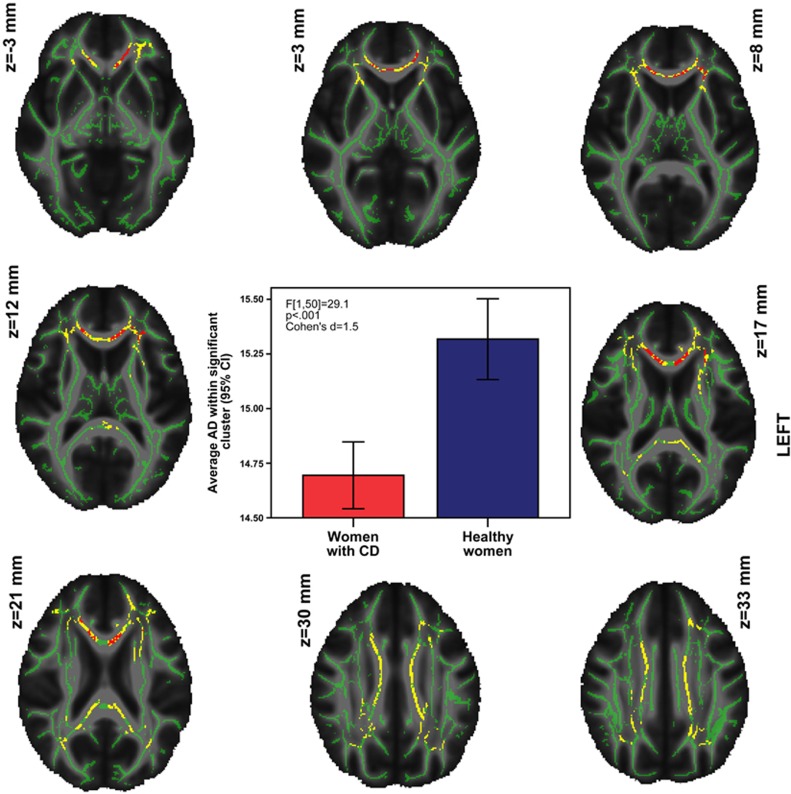
Reduced axial diffusivity (AD) in women with conduct disorder (CD) compared with healthy women. Yellow voxels: reduced AD among women with CD compared with healthy women. Red voxels: reduced AD among women with CD after covarying for alcohol dependence, drug dependence, anxiety disorders, depression disorders, physical abuse and sexual abuse (cluster averages graphed). Overlaid on the fractional anisotropy (FA) skeleton (in green) and the mean FA image. AD values scaled by a factor of 10^4^ for sake of presentation.

Voxelwise analyses revealed a large area of decreased AD (13 469 voxels) in the women with CD, covering many regions of intersecting tracts, making tract identification difficult. The Johns Hopkins University tractography and region atlases^66, 67, 68^ indicated that this area (with any probability) included the genu, body and splenium of the corpus callosum (including forceps minor and major), corona radiata, anterior thalamic radiation, corticospinal tract, cingulum, inferior fronto-occipital fasciculus, inferior longitudinal fasciculus, superior longitudinal fasciculus and UF, bilaterally. Tractography performed on a single subject confirmed that these tracts passed through the cluster (Supplementary material). Averaging AD values across the entire region and comparing groups revealed a large effect size (F[1,50]=37.17, *P*<0.001, Cohen's *d*=1.7 (95% confidence interval (CI): 0.87—2.35)). The women with CD, as compared with HC women, exhibited no areas of increased AD, nor any differences in FA or RD. Results are presented in Figure 1.

Re-running the significant contrast confined to the mask of voxels showing an AD difference removed 53% of the previously significant voxels when covarying for alcohol dependence, 14% covarying for drug dependence, 63% covarying for anxiety disorders, 74% covarying for depression disorders, 24% covarying for physical abuse and 82% covarying for sexual abuse. Conjunction analyses of the resulting statistical maps revealed that only a fraction of voxels (*n*=1164) remained significant in each and every contrast, as shown in red in Figure 1. These voxels covered primarily the body and genu (including forceps minor) of the corpus callosum, with some additional voxels covering bilateral cingulum, and the intersection of the left corona radiata and inferior fronto-occipital fasciculus. The UF was also indicated by the atlas, although this was judged to be an artifact of TBSS skeletonization and overlapping atlas probability maps. Tractography and manual dissection of the uncinate performed on a single subject corroborated that the uncinate did not pass through the surviving cluster (Supplementary material). The effect size of the average group difference in this surviving cluster was large (F[1,50]=29.1, *P*<0.001, Cohen's *d*=1.5 (95% CI: 0.82—2.12)). Because previous studies have shown that corpus callosum integrity is associated with IQ,^69^ *post hoc* analyses were computed. No significant correlations between average AD in the corpus callosum cluster and VIQ or PIQ were detected.

### Women with CD compared with a CC group

As presented in Table 1, compared to the clinical comparison group, women with CD were similar in age, obtained similar VIQ and PIQ scores, had similar handedness scores, and similar proportions in both groups acquired diagnoses (both lifetime and current) of alcohol and drug dependence, anxiety and depression disorders, and reported physical and sexual abuse. Fewer of the CD than CC women had graduated from high school and had a stable occupation, and there was a trend toward the CD group showing more recent aggressive behavior (*P*=0.078).

**Figure 2:**
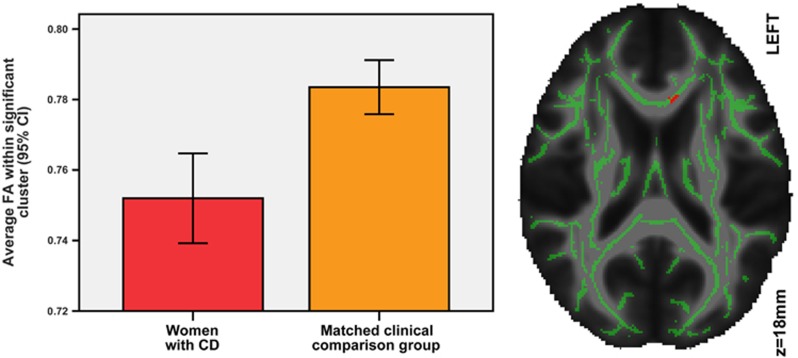
Reduced fractional anisotropy (FA) in women with conduct disorder (CD) compared with those in the clinical comparison group. Right: red voxels show reduced FA among women with CD compared with those in the clinical comparison group. Overlaid on the FA skeleton (in green) and the mean FA image.

Voxelwise comparisons between CD and CC groups were restricted to the conjunction mask of voxels that had shown decreased AD after correction for all confounders in the comparison of CD and HC groups. Only near significant reductions in AD were found (at best *P*_FWE_=0.22). However, significant reductions in FA among the women with CD were found in a cluster of 68 voxels covering primarily the genu and body of the corpus callosum (including the forceps minor), as shown in Figure 2. The effect size based on cluster-average FA values was Cohen's *d*=1.13 (95% CI: 0.6—1.71, F[1,41]=12.49, *P*=0.001 (survived Welch correction for unequal variance)). There were no correlations with age, VIQ, PIQ or handedness. To investigate the specificity of the FA abnormality to CD, the CD and CC groups were combined and the extracted cluster-average FA values of participants with and without each comorbid lifetime diagnoses and physical and sexual abuse were compared. No differences in the extracted cluster-average FA values were detected in comparisons of women with and without alcohol dependence (F[1,41]=2.09, *P*=0.156), anxiety disorders (F[1,41]=0.412, *P*=0.525), depression disorder (F[1,41]=0.605, *P*=0.441), physical abuse (F[1,41]=2.514, *P*=0.121) or sexual abuse (F[1,41]=0.864, *P*=0.358). Women with drug dependence, as compared with those without, showed reduced FA within this cluster (F[1,41]=6.703, *P*=0.013, Cohen's *d*=0.84 (95% CI: 0.08—1.60)). There were no significant differences between groups in RD within the conjunction mask.

## Discussion

We believe we show for the first time that young women who presented CD as adolescents displayed widespread reductions in AD, primarily in frontotemporal regions, relative to healthy women. Using two methods to take account of comorbid disorders and maltreatment, results indicated that CD was specifically associated with abnormalities of the corpus callosum. Only three of the women with prior CD met diagnostic criteria for ASPD. Yet, they showed several characteristics associated with antisocial behavior including failure to graduate from high school, low levels of adult psychosocial functioning, more frequent aggressive behavior and having a baby at a young age.^4, 70^

Unlike previous DTI studies of adolescents with CD, we systematically investigated the confounding effects of comorbid disorders and maltreatment that are common to CD. Two complementary analyses showed that abnormalities of the corpus callosum were specifically associated with CD, independent of confounders. The only difference between the women with CD and HC that remained significant after taking account of all confounders was decreased AD primarily in the forceps minor and genu of the body of the corpus callosum. Abnormalities of the corpus callosum have been reported in several studies of both adolescents with CD and adults with ASPD (who by definition present CD before the age of 15). Boys and girls with CD, compared with healthy peers, showed reduced AD in the forceps minor and corpus callosum.^24^ Boys with CD and no comorbid disorders, compared with healthy boys, showed increased FA in the genu and body of the corpus callosum.^25^ Adult men with ASPD, relative to healthy men, exhibited decreased FA in widespread frontal regions including the genu,^26^ and increased callosal volume and length, reduced callosal thickness and increased interhemispheric connectivity, after statistically adjusting for IQ and SUDs.^71^

The results of the present study confirm and extend previous findings by showing that CD in females is also associated with abnormalities of the corpus callosum and that these abnormalities are present in adulthood, even in the absence of a diagnosis of ASPD, and after controlling for IQ, alcohol and drug dependence, anxiety, depression and physical and sexual abuse. Thus, previous findings associating corpus callosum abnormalities with ASPD may in fact reflect an abnormality of development that is specifically associated with CD. Whereas reduced FA is an unspecific marker of white matter abnormalities, the additional finding of reduced AD suggests an abnormality of axonal structure (increased axonal tortuosity, axonal thinning or axonal loss) rather than of myelination.^72^ The FA metric incorporates the same primary eigenvalue that constitutes the AD metric, and is also sensitive to perpendicular eigenvalues. Even subtle (nonsignificant) group differences in perpendicular eigenvalues may amplify AD differences, manifesting as a difference in FA. Finding reduced FA in a region showing abnormal AD in another contrast featuring the same group is thus indicative of the same underlying abnormality. The same pattern of results, reduction of FA and AD but not RD, has previously been reported in a mixed sex sample that did not distinguish between boys and girls with CD.^24^

The corpus callosum is the largest fiber bundle in the brain, connecting the two cerebral hemispheres. The forceps minor forms an important subregion, passing through the genu of the corpus callosum to connect medial and lateral frontal cortices. There is an unresolved question of whether the primary function of the corpus callosum is to inhibit or excite contrahemispheric activity, aiding either functional lateralization or interhemispheric integration, or both.^73, 74^ Reduced AD and FA of the corpus callosum likely impedes interhemispheric communication, thereby compromising behaviors that are reliant on the integration of lateralized functions. Evidence from neuroimaging and neuropsychological studies suggests frontal asymmetry in the human motivational system, such that approach motivation, the excess of which may fuel impulsivity and anger, is lateralized to the left hemisphere, and avoidance motivation, the excess of which may lead to depression and anxiety, to the right hemisphere, with healthy functioning relying on successful interhemispheric inhibition of contralateral activity.^75^ Connectivity abnormalities of the corpus callosum, and in particular the forceps minor, may thus reflect interhemispheric imbalance. This imbalance may promote approach behaviors such as impulsivity and aggression due to inefficient inhibition of left hemisphere-based approach behaviors by right hemisphere activity. In line with this hypothesis, aggressive behavior is more common among males than females,^76^ and males show higher within-hemisphere connectivity and females higher between-hemispheric connectivity.^14^ Interhemispheric imbalance could also contribute to the increased prevalence of anxiety and depressive disorders (that is, avoidance-motivated behaviors) common among individuals with CD, assuming that left hemisphere activity is similarly inefficient in inhibiting right hemisphere activity.^77^ However, no differences in the corpus callosum were found between participants with and without lifetime histories of depression and anxiety disorders, suggesting that the corpus callosum abnormality is indeed specific to CD. An alternative, or perhaps, additional hypothesis is that abnormalities of the corpus callosum in our CD sample may reflect difficulty identifying the thoughts and emotions of others and the ability to integrate information from multiple sources.^78^ Similar to those with agenesis of the corpus callosum,^78^ children with CD show deficits in social cognition.^79^

Results of the present study suggest that CD in females is not associated with abnormalities of the UF that have previously been reported among boys with CD and men with ASPD. This finding concurs with results from the only previous study of females with CD.^18^ Considering the sex differences in the CD phenotype,^80^ healthy white matter architecture^81, 82^ and development,^15, 16^ volumes of total gray versus white matter^83^ and of orbitofrontal regions,^13^ it is indeed plausible that the neural correlates of CD differ in females and males. The UF abnormality may be primarily associated with aggressive behavior that is less prevalent among girls than boys with CD. In the present study, the negative uncinate finding could have resulted from insufficient power, further complicated by the fact that this tract is located in a region of many intersecting tracts.

As hypothesized, most of the abnormalities in white matter integrity exhibited by the women with prior CD were associated with comorbid disorders and maltreatment. These results are in line with previous findings showing reduced structural integrity associated with alcohol dependence, drug dependence, depression and anxiety disorders, and childhood maltreatment.^31, 32, 33, 34, 35, 36, 37, 38, 39, 40, 41, 42, 43, 44, 45^ Our findings highlight the importance of diagnosing comorbid disorders and assessing maltreatment when studying CD and of disentangling the abnormalities associated with each of these disorders and with maltreatment from the abnormalities associated with CD. As we and others have shown,^49, 84^ in the great majority of cases, CD onsets before anxiety and depression and SUDs. Thus, the neural mechanism underlying CD may render children with CD vulnerable to comorbid disorders and to the neural abnormalities associated with each of these disorders. Consequently, identifying the time during development when neural mechanisms underlying CD emerge is the key to developing interventions that change these mechanisms, and thereby reduce conduct problems, before the onset of the comorbid disorders.

The present study showed that, despite the fact that most of the women with prior CD did not have a diagnosis of ASPD, they showed abnormalities of white matter integrity associated with CD that had onset during early adolescence. Given the key role these women have in the intergenerational transfer of antisocial behavior,^5^ the costs that they incur to the health and social service systems,^85^ and the suffering they endure and impose on those around them, it is essential to prevent CD and the underlying brain abnormalities. To be effective, prevention needs to target women with prior CD beginning during pregnancy in order to limit the use of alcohol and drugs and provide good prenatal care and continue after the birth of child to avoid suboptimal parenting.^5^ An additional component to prevent CD is strengthening laws and public policies aimed at eliminating physical and sexual abuse of children, as it is known that maltreatment interacts with specific genotypes in females to increase the risk of delinquency and criminality.^11, 86^ Providing evidence-based parent training for parents of young children with CD reduces conduct problems.^87^ Future studies are needed to ascertain whether reductions in conduct problems resulting from improved parenting skills are associated with reductions in alterations of white matter tracts and healthy neural development.

### Limitations

Although larger sample sizes would be preferable, this is the second largest whole-brain DTI study of CD to date and the only one to include only females. Stringent statistical thresholds and large effect sizes add confidence to our findings. Although IQ shows considerable stability from childhood to late life,^88^ additional validity would have been gained by re-assessing IQ in CD and CC groups before scanning. The skeletonization procedure within TBSS improves intersubject registration, but decreases anatomical specificity.^89^ Thus, although our pattern of results suggests abnormalities of a specific tract (for example, the forceps minor), we cannot exclude the possibility that abnormalities of adjacent tracts (for example, anterior cingulum) contributed to the observed effects. Because tracts intersect, no attempt was made to limit overlap of probability-based atlas maps, yet this also limits inferences about specific tracts. In addition, the tensor model used to fit the diffusion data is vulnerable to intravoxel crossing fibers. However, our main findings were located in the corpus callosum body and forceps minor, where fiber directionality is very coherent. Future studies featuring tractography-derived tract dissections and advanced mapping techniques such as spherical deconvolution^90^ will be required to investigate specific tracts. Finally, there is no ideal strategy for disentangling comorbid conditions.^48^ Although common practice, statistically covarying for confounding variables related to the non-random grouping variable is precarious. For this reason, we also compared our CD group to a group of women without CD who presented similar proportions with comorbid disorders and experiences of physical and sexual abuse.

## Conclusions

Young adult women who had presented CD before the age of 15 showed widespread reductions in AD primarily in frontotemporal regions relative to healthy women. By adjusting comparisons of CD and healthy women for comorbid disorders and maltreatment, and by comparing CD women to a clinical sample with comorbid disorders but not CD, we showed for the first time that abnormalities of the corpus callosum, but not the uncinate or other frontotemporal regions, were specifically associated with CD. Although previous studies have identified abnormalities of the corpus callosum among boys with CD and men with ASPD, they did not control for comorbid disorders or maltreatment. Taken together, the results of the present and previous studies suggest that abnormalities of the corpus callosum may be a developmental biomarker of antisocial behavior among both females and males that remain through adulthood even in the absence of ASPD. Contrary to findings in males with CD, women with CD did not exhibit anomalies in the uncinate. Comorbid disorders and maltreatment that are common in CD were each associated with abnormalities of white matter integrity.

## Figures and Tables

**Table 1:** Sample characteristics

*Variable*	*CD*	*HC*	*CC*	*Statistics CD versus HC*	*Statistics CD versus CC*
Mean (s.d.) age	24.1 (2.9)	22.9 (3.4)	25.2 (1.6)	F(1,50)=1.79, *P*=0.187	F(1,41)=1.7, *P*=0.2
Mean (s.d.) handedness score	87.6 (33.6)	73.0 (60.4)	92.0 (10.3)	W=305, *P*=0.72	W=219, *P*=0.82
Mean (s.d.) verbal IQ	7.9 (2.3)	9.5 (1.6)	8.1 (1.8)	F(1,49)= 7.5, *P*=0.009	F(1, 40)=0.09, *P*=0.77
Mean (s.d.) performance IQ	8.6 (3)	9.9 (1.7)	9.9 (2.6)	F(1,49)=3.46, *P*=0.069	F(1, 40)=1.99, *P*=0.166
% Completed high school	42.9%	79.2%	86.7%	FET *P*=0.011	FET *P*=0.009
Mean (s.d.) number of lifetime max CD symptoms	6.0 (3.0)	0 (0)	0.5 (0.5)	F(1,50)=97, *P*<0.001	F(1,41)=50.29, *P*<0.001
Indication of ADHD	10.7%	0%	0%	FET *P*=0.24	FET *P*=0.541
% Antisocial personality disorder	12%	0%	0%	FET *P*=0.235	FET *P*=0.279
					
*Lifetime diagnoses*					
% Alcohol dependence	46.4%	0%	26.7%	FET *P*<0.001	FET *P*=0.327
% Drug dependence	39.3%	0%	20.0%	FET *P*<0.001	FET *P*=0.308
% Any anxiety disorder	82.1%	0%	73.3%	FET *P*<0.001	FET *P*=0.696
% Any depression disorder	75.0%	0%	73.3%	FET *P*<0.001	FET *P*=1
					
*Diagnoses at MRI*					
% Alcohol dependence	3.6%	0%	0%	FET *P*=1	FET *P*=1
% Drug dependence	7.1%	0%	0%	FET *P*=0.493	FET *P*=0.535
% Any anxiety disorder	28.6%	0%	13.3%	FET *P*=0.005	FET *P*=0.451
% Any depression disorder	14.3%	0%	0%	FET *P*=0.115	FET *P*=0.280
					
*Exposure to maltreatment*					
% Physical abuse by parents	35.7%	0%	20%	FET *P*<0.001	FET *P*=0.487
% Sexual abuse	64.3%	0%	46.7%	FET *P*<0.001	FET *P*=0.338
					
*Psychosocial functioning in adulthood*					
% Full-time stable occupation last 12 months	46.2%	100%	85.7%	FET *P*<0.001	FET *P*=0.02
% With children	51.9%	13%	26.7%	FET *P*=0.006	FET *P*=0.193
Self-reported aggressive behaviors last 6 months (s.d.)	0.93 (1.7)	0.12 (0.5)	0.13 (0.4)	F(1,50)=5.18, *P*=0.027	F(1,41)=3.26, *P*=0.078

Abbreviations: AD, axial diffusivity; ADHD, attention-deficit hyperactivity disorder; CC, clinical comparison; CD, conduct disorder; FET, Fisher's exact test; HC, healthy comparison; IQ, intelligence quotient; MRI, magnetic resonance imaging; W, Wilcoxon rank sum statistic.
