# Supplementary material for: Conduct disorder in females is associated with reduced corpus callosum structural integrity independent of comorbid disorders and exposure to maltreatment
Source: Transl Psychiatry. 2016 Jan 19;6(1):e714–. doi: 10.1038/tp.2015.216 (PMC5068887; doi:10.1038/tp.2015.216)
Supplement: Supplementary Information [file tp2015216x1.pdf]

Supplementary material for:

Conduct disorder in females is associated with reduced corpus callosum structural integrity independent of comorbid disorders and exposure to maltreatment

Philip Lindner[1,2]

Ivanka Savic[3,4]

Rouslan Sitnikov[1]

Meenal Budhiraja[1,2]

Yawu Liu[5,6]

Jussi Jokinen[1,2,7]

Jari Tiihonen[1,2,8,9]

Sheilagh Hodgins[1,10]

[1]Department of Clinical Neuroscience, Karolinska Institutet, Stockholm, Sweden

[2]Centre for Psychiatry Research, Karolinska Institutet, Stockholm, Sweden

[3]Department of Women's and Children's Health, Karolinska Institutet, Stockholm, Sweden

[4]Neurology Clinic, Karolinska University Hospital, Huddinge, Sweden

[5]Department of Clinical Radiology, Kuopio University Hospital, University of Eastern Finland, Kuopio, Finland

[6]Department of Neurology, Kuopio University Hospital, University of Eastern Finland, Kuopio, Finland

[7]Department of Clinical Sciences, Umeå University, Umeå, Sweden

[8]Department of Forensic Psychiatry, University of Eastern Finland, Niuvanniemi Hospital, Kuopio, Finland

[9]National Institute for Health and Welfare, Helsinki, Finland

[10]Département de Psychiatrie, Université de Montréal, Montréal, QC, Canada

Corresponding author:

Philip Lindner

Psychiatry Building R5:00, Karolinska Universitetssjukhuset, 171 76, Stockholm, Sweden

philip.lindner@ki.se

Supplementary Methods

Supplementary Table S1. Clinical measures details

**Measure**	**Details**
Alcohol dependence	Life-time diagnosis of alcohol dependence, presence or absence
Drug dependence	Life-time diagnosis of drug dependence, presence or absence
Any anxiety disorder	Life-time diagnosis of agoraphobia, generalized anxiety disorder, anxiety disorder not-otherwise-specified, obsessive compulsive disorder, panic disorder, post-traumatic stress disorder, social phobia, specific phobia or substance-induced anxiety disorder, presence or absence
Any depressive disorder	Life-time diagnosis of major depressive disorder, dysthymia, depressive disorder not-otherwise-specified or substance-induced mood disorder, presence or absence
Physical abuse	Hit with a fist or kicked hard, hit on a part of the body other than the bottom with a hard object, thrown or knocked down, grabbed around the neck and choked, beaten up, hit repeatedly very hard, burned, or threatened with a gun or knife, according to answers to the Conflict Tactics Scales, presence or absence
Sexual abuse	At any time in life forced to have sex against her/his will by a person in position of authority, by offering alcohol or drugs, or by physical violence, according to the Sexual and Physical Abuse Questionnaire, Sexual Experience Survey or MacArthur Community Violence Instrument.
Indication of ADHD	Self-report of at any time in life having received a diagnosis of ADHD or

	being prescribed methylphenidate. For the CC and CD groups, diagnoses from the National Swedish Health Register from the last assessment wave were also available.
% Full-time stable occupation past 12 months	At least 11 months of full-time activity (work, education, job-training or parental leave), during the past 12 months, recorded using the Life History Calendar (LHC).
Self-reported aggressive behaviors last 6 months	Number of aggressive behaviors against others as reported during an interview using the MacArthur Community Violence Instrument.

MRI preprocessing

Automated quality control and correction of images was performed using DTIPrep (Oguz et al., 2014). DTIPrep identifies and removes low-quality volumes and corrects all remaining volumes for motion and eddy-currents (including gradient direction adjustment). The three groups did not differ in number of volumes excluded due to poor quality (mean number of volumes excluded=1.67; Kruskal-Wallis χ^2=3.2[2], p=.21).

Further pre-processing was carried out using the FSL software package (Jenkinson et al., 2012; Smith et al., 2004). Tensor-fitting was performed using the *dtifit* tool and weighted least-squares regression, generating fractional anisotropy (FA) and axial (AD) and radial diffusivity (RD) maps. Scans were conducted during a period of 18 months, during which a minor scanner update was made. To ensure that this update did not impact results, protocol version (0 or 1, demeaned across entire sample) was included as a covariate of no interest in all TBSS analyses mentioned in the main manuscript (along with other possible covariates), as frequently done in multi-center studies. We also re-ran the primary between-group analyses including only the participants scanned with the first protocol (see Supplementary Figure S1 below). Since the obtained results were nearly identical to those that included the full sample, we concluded that the protocol update did not affect our analyses and used the full sample.

Controlling for scanner protocol update

Prior to any preprocessing, nifty files for all participants scanned with old protocol were re-sampled to the dimensions of the new protocol using the AFNI script *3dresample* and the interpolate method. The primary analysis was re-run using only subjects scanned with the old protocol: 23 women with a history of conduct disorder (CD), 11 healthy women and 14 women from the clinical comparison group. Using this subsample, we were able to replicate the findings

of widespread reductions in axial diffusivity (AD) in women with CD compared to healthy women. See Figure S1 below.

Tractography

In order to visually confirm which tracts run through the large cluster of decreased AD observed among the women with CD compared to healthy women, tractography was performed on a randomly chosen participant. First, the cluster was de-projected into the participant's native space using the *tbss_deproject 2* script. The preprocessed image was then independently tensor-fitted and whole-brain tractography performed using Diffusion Toolkit. Tractography was performed using the interpolated streamline algorithm, an angle threshold of 34° and an FA threshold of 0.2-1, similar to a recent tractography study of psychopathy (Sethi et al., 2014). In Trackvis, all tracks passing through the cluster were visualized. See Figure S2 below.

Using the same tractography, we also visualized the tracts running through the cluster of decreased AD observed in women with CD compared to healthy women that survived adjusting for all lifetime psychiatric disorders and experience of maltreatment. Since the atlas probability maps indicated some probability of the surviving cluster covering the uncinate pathway, and this tract has been consistently implicated in antisocial behavior and psychopathy, we independently dissected the left uncinate manually using the same validated dissection protocol used in Sethi et al. (2014). The uncinate was then visualized alongside the surviving cluster (de-projected into native space). See Figure S3. Results corroborated that the uncinate did not pass through the significant cluster.

References

Jenkinson, M., Beckmann, C.F., Behrens, T.E.J., Woolrich, M.W., Smith, S.M., 2012. FSL. Neuroimage 62, 782–90. doi:10.1016/j.neuroimage.2011.09.015

Oguz, I., Farzinfar, M., Matsui, J., Budin, F., Liu, Z., Gerig, G., Johnson, H.J., Styner, M., 2014. DTIPrep: quality control of diffusion-weighted images. Front. Neuroinform. 8, 4. doi:10.3389/fninf.2014.00004

Sethi, A., Gregory, S., Dell'Acqua, F., Periche Thomas, E., Simmons, A., Murphy, D.G.M., Hodgins, S., Blackwood, N.J., Craig, M.C., 2014. Emotional detachment in psychopathy: Involvement of dorsal default-mode connections. Cortex. 1–9. doi:10.1016/j.cortex.2014.07.018

Smith, S.M., Jenkinson, M., Woolrich, M.W., Beckmann, C.F., Behrens, T.E.J., Johansen-Berg, H., Bannister, P.R., De Luca, M., Drobnjak, I., Flitney, D.E., Niazy, R.K., Saunders, J., Vickers, J., Zhang, Y., De Stefano, N., Brady, J.M., Matthews, P.M., 2004. Advances in functional and structural MR image analysis and implementation as FSL. Neuroimage 23 Suppl 1, S208–19. doi:10.1016/j.neuroimage.2004.07.051

Figure S1. CD< HC (p_{FWE}<0.0167) differences in AD with full vs. subsample

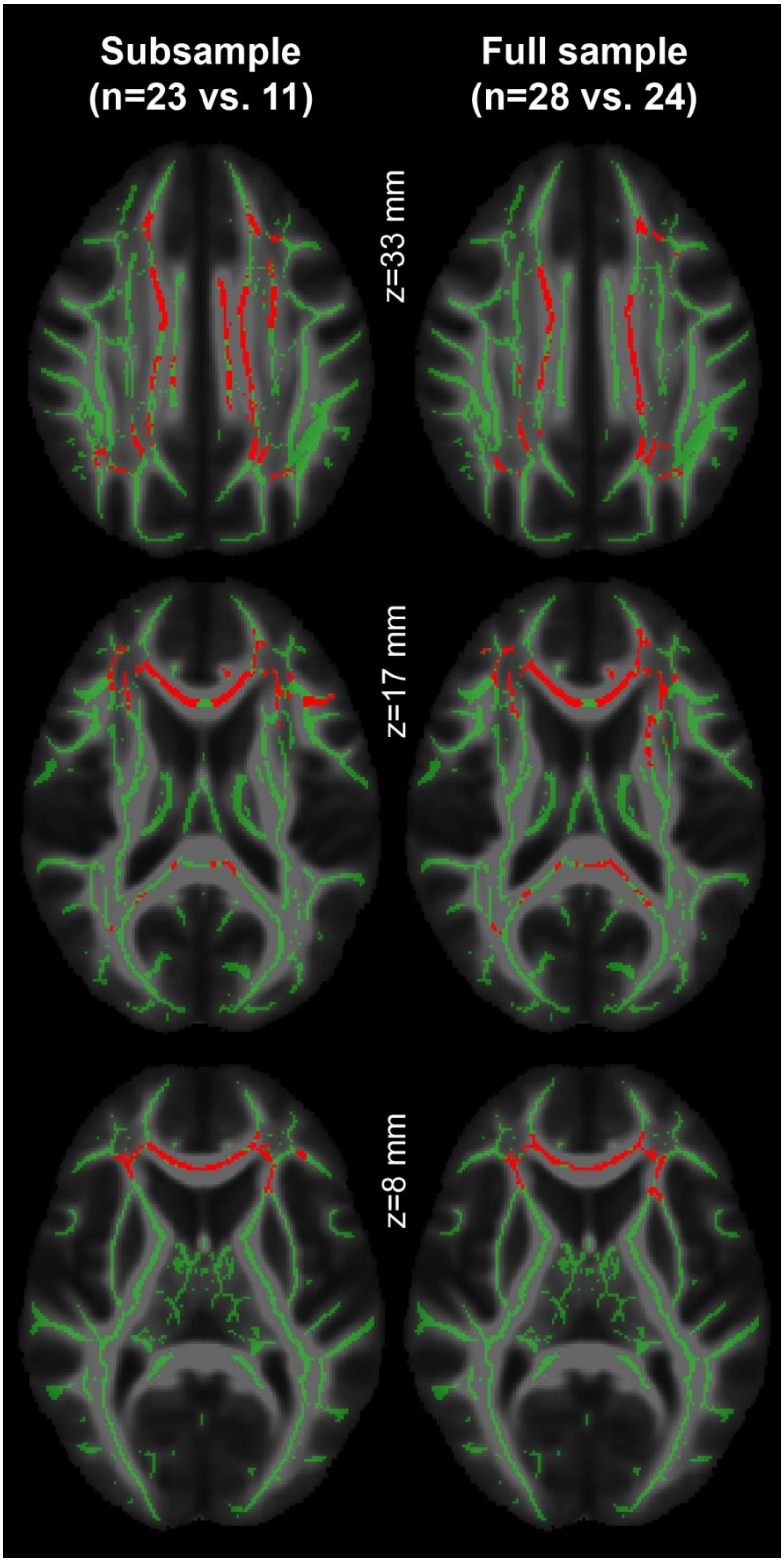

Left hemisphere to the right.

Figure S2. Tracts running through voxels with significant CD<HC differences in AD

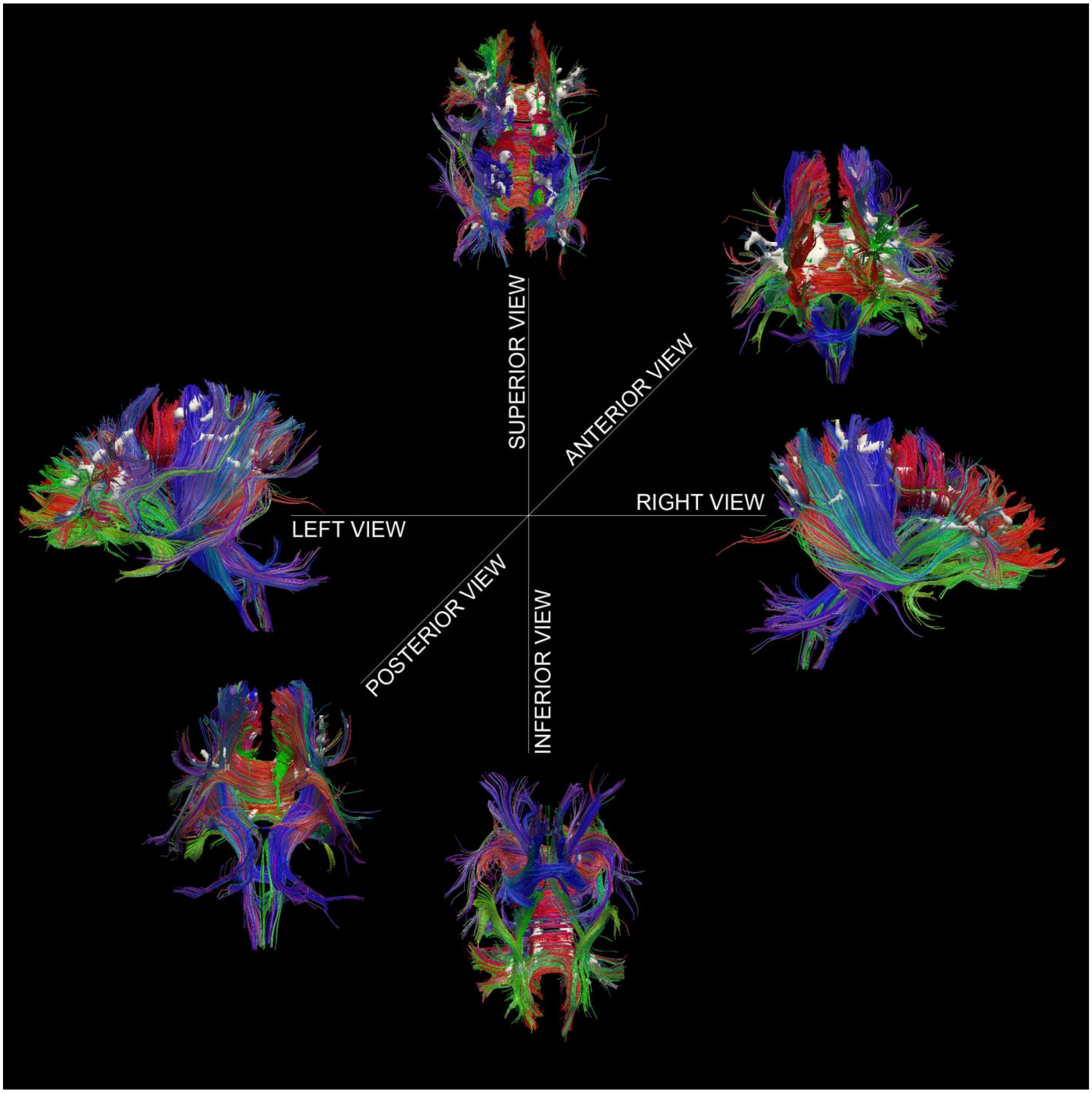

Whole-brain tractography performed on a single subject with significant cluster (in white) as ROI. Non-implicated tracts not shown. Tracts colored according to direction. Upper tract length threshold of 144 mm applied to remove tractography artefacts.

Figure S3. Tracts running through voxels with significant CD<HC differences in AD after correction for all confounders, alongside the uncinate fasciculus

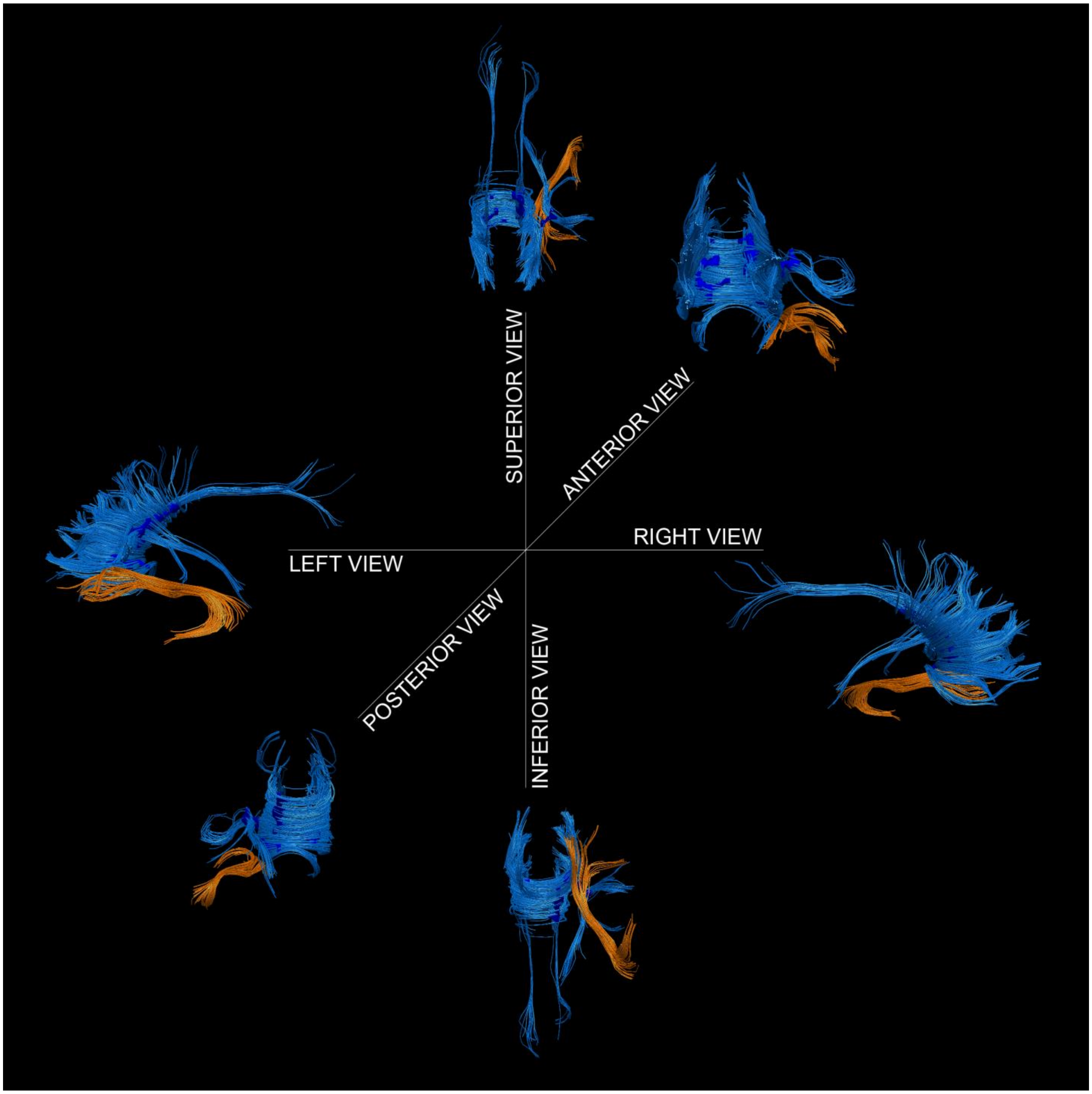

Whole-brain tractography performed on a single subject. In light blue, tracts passing through the significant surviving CD<HC cluster (in dark blue). In orange, the left uncinate fasciculus, manually dissected. Non-implicated tracts not shown.
